# Palliative Radiotherapy for Disfiguring Mycosis Fungoides Lesion: A Key Treatment to Reduce Psychological and Social Impact

**DOI:** 10.1155/2018/1762050

**Published:** 2018-07-26

**Authors:** Axel Egal, Caroline Ram-Wolf, Laurent Quero, Martine Bagot, Marie-Dominique Vignon-Pennamen, Christophe Hennequin, Basma M'Barek

**Affiliations:** ^1^Department of Radiotherapy, Montfermeil Hospital, France; ^2^Department of Dermatology, Saint Louis Hospital, AP-HP, Paris, France; ^3^Department of Radiotherapy, Saint Louis Hospital, AP-HP, Paris, France; ^4^Department of Pathology, Saint Louis Hospital, AP-HP, Paris, France

## Abstract

Mycosis Fungoides (MF) is a rare disease with a relatively good prognosis at early stage. However, skin lesions can impair quality of life due to extensive skin lesions. In some cases, skin lesions, and especially those of the face, become visible and change the physical appearance of the patients. This aspect can deeply affect patients psychologically and can impact their social life. Here, we report the case of a patient with multiple lesions including a disfiguring lesion arising from the nose. The extent of his skin lesions gave a palliative intent to his treatment project. The patient underwent several lines of chemotherapy and immunotherapy with poor results. He was then referred to our radiotherapy and received localized radiotherapy. Lesions disappeared completely within a few weeks. The patient reported a psychological relief. This case highlights the fact that radiotherapy can be done in a “palliative” intent in order to improve esthetic aspects of lesions that can dramatically impact the psychosocial side of patient's life. Clinicians can consider radiotherapy as treatment of some MF lesions as far as they impair the patient's comfort from a psychological and social point of view.

## 1. Introduction

Mycosis Fungoides (MF) is the most common form of cutaneous T-cell lymphoma. It typically affects patients with a median age of 55 to 60 years, mostly men [1]. The disease is traditionally indolent at early stage and progresses slowly. However, both folliculotropic variant and large cell transformation are known to have worse outcomes.

Skin involvement is classically presenting as pruritic erythematous patches in nonsun exposed areas that can evolve toward infiltrative plaques and tumors. Overall survival is usually very good at early stage with reported rates of 5-year survival approaching 90% [2] and thus treatment is primarily determined by disease extent and impact on quality of life. Multiple treatments exist for early MF such as topical corticosteroids, topical chlormethine, or phototherapy [3]. In case of extensive or more aggressive disease, systemic treatments can be introduced such as retinoids, interferon, methotrexate, histone deacetylase inhibitors, monoclonal antibodies, systemic chemotherapy, total skin electron beam therapy (TSEBT), and sometimes even allogeneic stem cell transplantation [4, 5]. Local radiation therapy may be useful in selected patients with localized infiltrative and/or ulcerating cutaneous lesions. Complete and long-lasting response rates reported in the literature are often higher than 90 percent [6–9]. Several treatment schedules are possible with multifractionated doses being the most used. At the opposite, low-dose radiation with a single or 2 fractions of 7 or 8 Gy are also possible [8, 10]. Compared to TSEBT, local radiotherapy presents less toxicities and better tolerance [11].

## 2. Case Report

A Folliculotropic Mycosis Fungoides was diagnosed in a 58-year-old male patient in 1997 and treated with local chlormethine between 1998 and 2006.

In 2006, MF progressed toward a tumoral form with infiltrating plaques and nodules all over his body, the most important being an exophytic one arising from the nasal region. No Sezary cell was noted in the blood smear. A biopsy of cutaneous tumor was performed and the pathologist confirmed a localization of tumoral nontransformed MF.

**Figure 1:**
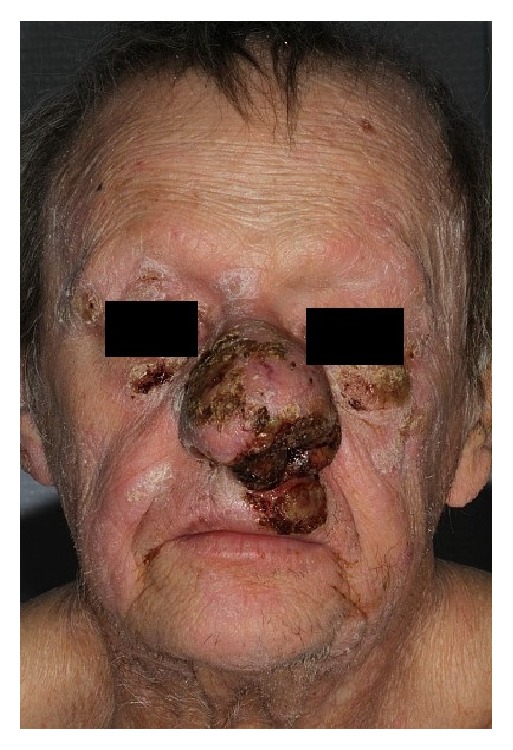
Exophytic lesion of the nose before radiotherapy (front view).

**Figure 2:**
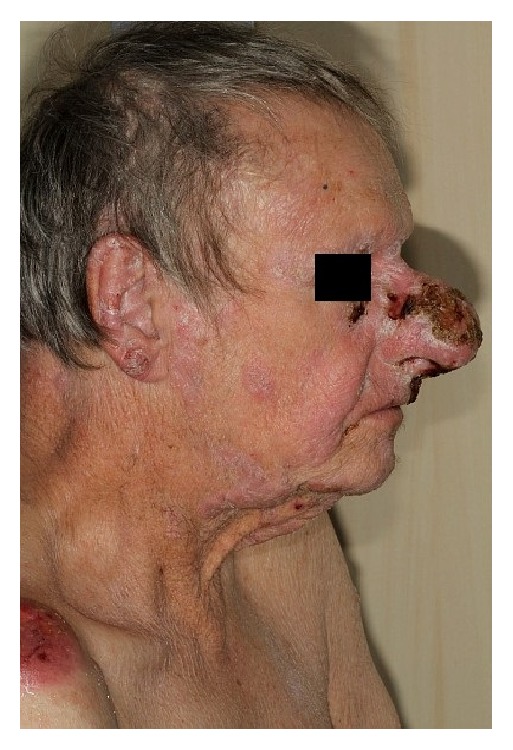
Exophytic lesion of the nose before radiotherapy (side view).

Between 2006 and 2014 the patient received several systemic treatment lines including methotrexate, PUVA therapy, pegylated liposomal doxorubicin, polychemotherapy, histone deacetylase inhibitors, and anti-CCR4 monoclonal antibody. All these drugs were without long-lasting effect and tumoral lesions progressed including the tumoral lesion of the nose (Figures 1 and 2).

The extent, progression, and resistance of his skin lesions gave a palliative intent to his treatment project. The patient reported that the aspect of his nose refrained him from interacting with people, which led him progressively to get socially isolated. He reported difficulties in interacting with his family members especially with his young grandchildren. Histology from the nasal lesion was obtained and showed classical Mycosis Fungoides of granulomatous type without transformation.

He was then referred to our radiotherapy unit in August 2014. We opted for a conventional radiotherapy with 12 MeV electrons and 6 MV and 18 MV photons. The patient received 36 Gy in 18 fractions (2 Gy per fraction, 5 fractions per week).

**Figure 3:**
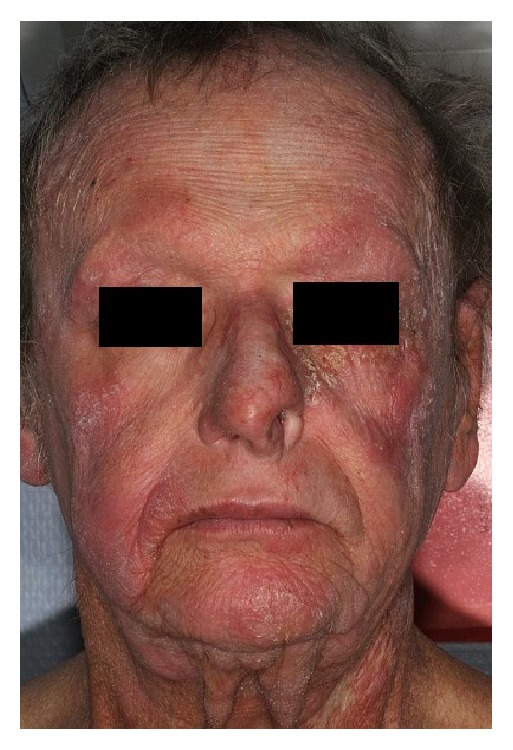
Aspect after radiotherapy (front view).

**Figure 4:**
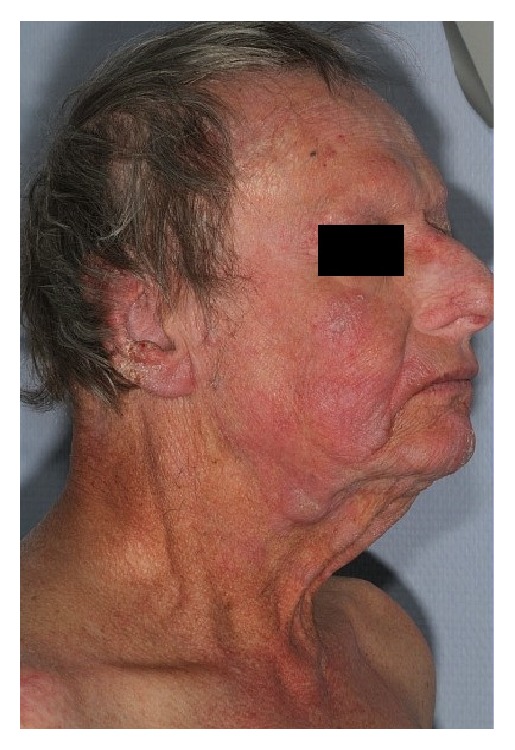
Aspect after radiotherapy (side view).

Lesions disappeared completely within a few weeks (Figures 3 and 4). The patient presented acute grade I radiodermatitis (NCI CTCAE Version 4.03) which resolved spontaneously. No clinical relapse had been noted 3 years after the treatment.

As the physical appearance of the irradiated nose got better the patient reported a psychological relief. The recovery of the normal aspect of his nose helped him resume some of his social activities, use public transportation, and better interact with friends and family members.

## 3. Discussion

Radiotherapy is a highly effective treatment option of MF, which can lead to excellent response rates when used in both curative and palliative intent. The high radio sensitivity of the MF lesions makes this treatment option possible in palliative intent because of the limited treatment options. The international lymphoma radiation group as well as the European Organization for Research and Treatment of Cancer (EORTC) includes radiotherapy as a key treatment option in both curative and palliative intent for MF skin lesions [11, 12].

This patient's case report highlights the fact that radiotherapy can also be done in a “palliative” intent, in order to improve quality of life in its psychological and social dimension. Lesions of the uncovered skin, especially lesions located on the face, may impact the patient's social life. Taking into account the psychological and social impacts of the disease is important. Radiotherapy can be of great help as it comes to improve the local aspect of the skin lesions. Indeed, most of the skin lesions have an excellent response rate to radiotherapy even if in the literature, lower responses rate are common in transformed MF and/or in lesions associated with poor circulation and wound healing located on the lower extremities. Moreover, radio-sensitizing agents, such as histone deacetylase inhibitors, are thought to work synergistically with low-dose local radiation therapy [13, 14].

Required dose varies with the intent of the treatment and tends to be more important in isolated unique lesion of MF. It has been reported a dose-dependent response with better local control with delivered doses around 30 Gy [7]. Irradiation is delivered to the macroscopic gross tumor volume with 1 cm margins. In patients treated with curative intent, margins tend to be larger (2 cm) for isolated unique lesion of MF. The European society for treatment and research for cancer advises to deliver doses around 24 Gy [12]. The international lymphoma radiotherapy oncology group suggests dose range between 24 and 30 Gy, as local recurrences seem to be rare when a minimal dose of 24 Gy is delivered [11]. Electron beam therapy is well adapted as well as orthovoltage radiation therapy (100 kV). For thicker lesions, as for the case we have reported, the use of higher energy photons (6 MeV) may be needed. Low-dose and short-course irradiation lead to better patient convenience even in a palliative setting.

Both the European consensus on the treatment and the international lymphoma radiation oncology group consider the treatment by total skin electron beam therapy as a treatment option. This radiotherapy technique consists in delivering low doses irradiation (10-12 Gy) to larger volumes, which has the benefits of being shorter in duration, with fewer side effects. Retreatment is even feasible if required. This treatment option is unfortunately not widely available and could not be delivered on a routine basis by most radiotherapy teams.

We used a 36 Gy total dose irradiation delivered in a classical regimen of 2 Gy/fraction, 5 fractions a week in order to ensure the best possible chances of local control while assuring a good treatment tolerance. Electron beam therapy is the most frequent way of doing described in the literature but in our case, nasal infiltration was so important that we added photons in order to achieve better lesion coverage. It is of interest to note that side effects were low despite the dose and that a major efficacy was obtained. As it is notable in the figures below, wounds of the nose healed and the nose walls got thinner offering a normal aspect for the nose's skin and mucosa.

Multidisciplinary boards with participation of psychologists, social workers as well as radiation oncologists, and palliative care actors are important in the comprehensive patient care of advanced stage of MF.

## 4. Conclusion

In conclusion, local radiotherapy is an effective treatment in infiltrated or tumoral lesions of MF and can be considered with a “palliative” intent in order to improve quality of life in patients with disfiguring lesions impacting their psychosocial life. Radiotherapy induces a high response rate with little toxicity and can be done concomitantly with other systemic treatments.
